# Fetal genetic findings by chromosomal microarray analysis and karyotyping for fetal growth restriction without structural malformations at a territory referral center: 10-year experience

**DOI:** 10.1186/s12884-023-05394-y

**Published:** 2023-01-26

**Authors:** Xiaoqing Wu, Shuqiong He, Ying Li, Danhua Guo, Xuemei Chen, Bin Liang, Meiying Wang, Hailong Huang, Liangpu Xu

**Affiliations:** 1 Medical Genetic Diagnosis and Therapy Center of Fujian Maternity and Child Health Hospital, College of Clinical Medicine for Obstetrics & Gynecology and Pediatrics, Fujian Medical University, No.18 Daoshan Road, Fuzhou City, Fujian, 350001 China; 2 Fujian Provincial Key Laboratory for Prenatal Diagnosis and Birth Defect, Fuzhou, Fujian China; 3 Department of Laboratory Medicine, Fujian Medical University, Fuzhou, Fujian China

**Keywords:** Fetal growth restriction (FGR), Cytogenetic karyotyping, Single nucleotide polymorphism array (SNP array), Maternal serum screening

## Abstract

**Background:**

Prenatal invasive genetic testing is commonly recommended to pregnancies of early-onset FGR or FGR combined with a structural defect. Our study aimed to explore the genetic findings for FGR without structural malformations according to cytogenetic karyotyping and single nucleotide polymorphism array (SNP array) technology over a 10-year period.

**Methods:**

A total of 488 pregnancies diagnosed with FGR without structural malformation were retrospectively reviewed. Cytogenetic karyotyping was performed on all the subjects, and SNP array was available from 272 of them. Based on the gestational age at onset, the cohort was classified into four groups: ≤ 24, 25–28, 29–32, and > 32 weeks of gestation. According to the ultrasound findings, they were grouped into isolated FGR, FGR with soft markers, and FGR with non-structural anomalies. In pregnancies of young maternal age, based on the results of maternal serum screening (MSS), they were categorized into high-risk and low-risk MSS groups.

**Results:**

Nineteen (3.9%) cases of chromosomal abnormalities were detected by cytogenetic karyotyping, including 11 cases of numerical abnormalities, 5 cases of structural abnormalities, and 3 cases of mosaicism. Trisomy 21 was the most frequent abnormality. Abnormal karyotypes were more frequently observed in cases diagnosed at ≤ 24 weeks (7.2%) than those in any other group. Among pregnancies with normal karyotype, an incremental yield of 4.2% were revealed by SNP array technology regarding clinically relevant aberrations. The additional detection rates by SNP array in cases diagnosed at ≤ 24 weeks (6.5%), cases with soft markers (9.5%), and cases with high-risk MSS (12.0%) were higher than those in other groups within each classification. All the cases with abnormal karyotypes and 7 out of 11 pregnancies with clinically relevant anomalies revealed by SNP array alone resulted in pregnancy terminations.

**Conclusion:**

Chromosome abnormality is an important etiology for FGR with no associated structural malformations, and plays a crucial role in pregnancies decision-making. SNP array improves the detection of genetic anomalies especially in FGR diagnosed at ≤ 24 weeks, FGR combined with soft makers, and FGR combined with high-risk MSS.

## Background

Fetal growth evaluation is essential for prenatal care and management. Fetal growth restriction (FGR) is a condition that the fetus fails to achieve genetic growth potential in utero [1, 2], which affects approximately 10% of pregnancies [2, 3]. It is commonly defined based on an estimated fetal weight (EFW) or abdominal circumference (AC) below the 10th percentile assessed by ultrasonography.

Multiple factors including maternal clinical conditions, genetic anomalies, and placental factors, have been reported to affect fetal growth in utero. The genetic etiology of FGR can be either fetal, maternal or placental [4], among which, the fetal genetic anomalies play an important role in pregnancy decision-making. Chromosomal abnormality detected using karyotyping has been estimated in 2–30% of fetuses with FGR [1, 5–8], with aneuploidy being the most frequent anomaly, accounting for about 5.8% in previous publications [9, 10]. It has been reported that FGR was present in at least 50% of fetuses with trisomy 13 or trisomy 18[11]. With the wide application of chromosomal microarray analysis (CMA), which is known to improve the detection of genomic abnormalities compared to cytogenetic karyotyping, more and more submicroscopic variants with clinical significance were observed in fetuses with FGR and normal karyotype. The incremental yield of significant copy number variants (CNVs) varied from 2.2%-10.1% [12–14]. According to the American College of Obstetricians and Gynecologists (ACOG) and the Royal College of Obstetricians and Gynecologists (RCOG), prenatal genetic diagnostic testing should be offered to pregnancies of mid trimester onset FGR or FGR accompanied with structural malformations[15]. With regard to the utility of CMA in FGR pregnancies, the Society for Maternal–Fetal Medicine (SMFM) recommends that women are offered CMA when FGR is associated with fetal malformation regardless of gestational age, or when unexplained isolated FGR is diagnosed before 32 weeks of gestation[16]. Here we presented our experience using karyotype analysis and Single nucleotide polymorphism array (SNP array) to explore the fetal genetic etiology of FGR without structural abnormalities.

## Materials and methods

### Patients and samples

This retrospective study included 488 singleton pregnancies that underwent invasive prenatal diagnosis in our center due to the diagnosis of FGR without apparently structural malformations during May 2011 and December 2021. Among them, cases with confirmed maternal medical conditions such as gestational diabetes mellitus, autoimmune diseases, pre-eclampsia, abnormal uterine artery Doppler were not included. In addition, pregnancies with Cytomegalovirus infection which was determined by cytomegalovirus DNA testing in prenatal samples were also excluded.

FGR was defined as Estimated Fetal Weight (EFW) or abdominal circumference (AC) below the 10th percentile based on the Hadlock formula.

The mean value of gestational age at FGR initially diagnosed was 26 ± 4.1 weeks. Based on the gestational age, the cohort was classified into four groups: ≤ 24, 25–28, 29–32, and > 32 weeks. According to the ultrasound findings, they were categorized into groups of isolated FGR, FGR accompanied with nonstructural abnormality including polyhydramnios, oligohydramnios and pleural effusion, and FGR accompanied by soft markers. Women of advance maternal age (AMA) and young maternal age (YMA) accounted for 13.7% (67/488) and 86.3% (421/488), respectively; the mean maternal age was 28.3 ± 4.6 years. In addition, information of maternal serum screening (MSS) from YMA pregnancies was also recorded for analysis.

The specimens included 275 cases of amniotic fluid sampled between 17 and 26 weeks of gestation, and 213 cases of umbilical cord blood sampled after 26 weeks of gestation. Cytogenetic karyotyping was performed in all 488 samples. Because SNP array was not applied in our center before June 2016, data of SNP array were available in 272 samples. The study was approved by the local Ethics Committee of Fujian Maternity and Child Health Hospital. All experiments were performed in accordance with relevant guidelines and regulations.

### Cytogenetic karyotyping

Cytogenetic karyotyping consisted of cell culture and G-banded karyotyping was performed according to the standard protocols in local laboratory. The karyotype was interpreted at a resolution of 320–500 bands level and was analyzed with international system for human cytogenetic nomenclature 2020 (ISCN 2020).

### SNP array and data interpretation

In 316 cases, uncultured amniotic fluid and cord blood were used for SNP array analysis after genomic DNA extraction. SNP array was performed using Affymetrix CytoScan 750 K array (Affymetrix Inc., Santa Clara, CA, UA), which includes 200,000 probes for single nucleotide polymorphisms and 550,000 probes for copy number variations (CNVs) distributed across the entire human genome. As described in our previous publication[17], microarray-based CNV analysis was performed using the Chromosome Analysis Suite software (ChAS), version 3.1 (Affymetrix, Santa Clara, CA, USA) and genomic imbalances were annotated based on the GRCh37/hg19 Genome Build (July 2013). A resolution was generally applied: gains or losses of ≥ 400 kb and regions of homozygosity (ROH) ≥ 10 Mb. Uniparental disomy (UPD) was reported based on the identification of the region of homozygosity (ROH) covering the entire chromosome. UPD tool was used for genome-wide detection of UPD within the child-parent trios to confirm maternal or paternal UPD origin. All detected CNVs were compared with in-house and national public CNV databases as follows: Database of Genomic Variants (DGV), Database of Chromosome Imbalance and Phenotype in Humans Using Ensemble Resources (DECIPHER), International Standards for Cytogenomic Arrays Consortium, and Online Mendelian Inheritance in Man (OMIM).

According to the American College of Medical Genetics (ACMG) definitions [18], CMA results were categorized into five levels: pathogenic, benign, likely pathogenic, likely benign, and variants of uncertain significance (VOUS). Pathogenic and likely pathogenic variants were considered clinically significant findings. Parental CMA was recommended to determine the inheritance of CNVs.

### Statistical analysis

The data were analyzed using SPSS software v26.0 (SPSS Inc., Chicago, IL, USA). Statistical comparisons were performed using the chi-square test, the Fisher's exact test, and p < 0.05 was considered statistically significant.

## Results

### Overall results of cytogenetic karyotyping and SNP array analysis

**Fig. 1:**
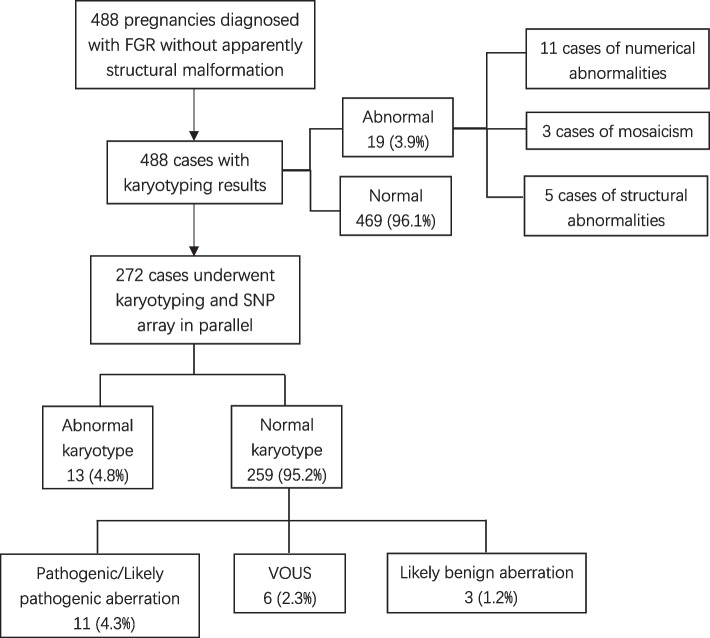
Summary of conventional karyotyping and SNP array results. VOUS, variants of uncertain significance

Summary of cytogenetic karyotyping and SNP array results was presented in Fig. 1. Nineteen (3.9%, 19/488) cases were found with abnormal karyotypes, including 11 cases of numerical abnormalities, 5 structural abnormalities, and 3 mosaicism. All these abnormal cases ended in termination of pregnancy (TOP). Trisomy 21 was the most frequent abnormality, accounting for 1.2% (6/488) of the cohort. The details are presented in Table 1.

**Table 1:** Clinical characteristics and chromosomal abnormalities detected by conventional karyotyping

Case number	Maternal age	Gestational age	Ultrasound findings	Karyotype	CMA results	Related syndrome	Outcomes
1	34	18	FGR, increased nuchal translucency, tricuspid regurgitation	47,XX, + 13	NA	Patau sydrome	TOP
2	25	20	FGR	47,XX, + 21	arr[GRCh37] (21) × 3	Down syndrome	TOP
3	26	19	FGR, increased nuchal fold, echogenic bowel	47,XX, + 21	arr[GRCh37] (21) × 3	Down syndrome	TOP
4	21	24	FGR	47,XX, + 21	NA	Down syndrome	TOP
5	29	20	FGR	47,XX, + 21	arr[GRCh37] (21) × 3	Down syndrome	TOP
6	29	18	FGR	47,XX, + 21	arr[GRCh37] (21) × 3	Down syndrome	TOP
7	32	19	FGR, increased nuchal fold, nasal bone dysplasia, echogenic bowel, tricuspid regurgitation	47,XY, + 21	arr[GRCh37] (21) × 3	Down syndrome	TOP
8	33	27	FGR	47,XXX	arr[GRCh37] (X) × 3	47,XXX syndrome	TOP
9	26	30	FGR	47,XXX	arr[GRCh37] (X) × 3	47,XXX syndrome	TOP
10	31	33	FGR	47,XXY	NA	Klinefelter syndrome	TOP
11	32	20	Oligohydramnios, Increased nuchal fold	47,XX, + mar dn	arr[GRCh37] 3p12.1p11.1(85,527,865–90,130,204) × 3,3q11.1q11.2(93,674,112–95,321,299) × 3 arr[GRCh37] 3p13p12.1(72,069,483–86,073,653) hmz,3q11.2q22.3(95,320,713–137,634,506) hmz		TOP
12	24	24	FGR, increased nuchal fold, aberrant right subclavian artery	46,XX,add(10)(q26)	arr[GRCh37]10q26.2q26.3(128,251,975–135,426,386) × 1,11q23.3q25(116,683,754–134,937,416) × 3		TOP
13	23	21	FGR	46,XX,der(9)ins(9;5)(q34.2;q35q34)mat	NA		TOP
14	27	23	FGR, increased nuchal fold	46,XX,dup(12)(q14q23)	arr[GRCh37] 12q14.2q23.1(64,877,459–97,710,202) × 3 dn		TOP
15	29	28	FGR	46,XY,add(7)(q35)	arr[GRCh37] 7q36.2q36.3(152,747,657–159,119,707) × 1, 11q23.1a25(111,067,572–134,937,416) × 3		TOP
16	29	23	FGR, mild ventriculomegaly, single umbilical artery, echogenic intracardiac focus	46,XY,del(4)(p15.3)	arr[GRCh37] 4p16.3p15.31(68,345–20,522,754) × 1	Wolf-Hirschhorn syndrome	TOP
17	30	21	FGR	46,X, + mar[54]/45,X[30]	NA	Turner syndrome	TOP
18	40	24	FGR, Oligohydramnios	47,XX, + psu idic(9)(q12)[39]/46,XX[11]	arr[GRCh37] 9p24.3q13(208,454–68,216,577) × 4		TOP
19	31	27	FGR, Single umbilical artery	47,XY + 8[27]/46,XY[23]	NA		TOP

*TOP* termination of pregnancy, *NA* not available

Among 272 cases underwent SNP array analysis in parallel, 259 showed normal karyotype. Of these cases with normal karyotype, 20 (7.7%) cases of abnormalities were revealed by SNP array, including 11 cases of pathogenic or likely pathogenic variants, 6 cases of VOUS, and 3 cases of likely benign variants, contributing to an incremental yield of 4.2% for clinically significant findings. As shown in Table 2, there were 9 cases of CNVs (cases 20–27) sized from 144 kb to 5.6 Mb, with 7 of them involving 22q11.2 microduplication syndrome (#608,363), Leri-weill dyschondrosteosis (LWD, # 127,300), Williams-Beuren syndrome (WBS, #194,050), Fanconi anemia, complementation group A (# 227,650), and X-linked Ichthyosis (# 301,800). Case 28 revealed neutral ROH of chromosome 6. Paternal uniparental disomy of chromosome 6 was confirmed after trio analysis using UPD tools. For case 29, amniotic fluid showed a normal karyotype, but low-level mosaicism of trisomy 2 was detected by SNP array. Subsequent cord blood sampled at the 28th gestational weeks revealed a maternal UPD(2). The fetus was live born, and trisomy 2 was confirmed in placental tissue by SNP array. The child had normally physical and mental development during a 3-year follow-up. Case 30 had positive results for trisomy 22 by non-invasive prenatal screening (NIPT), and the amniotic fluid sample at 21st gestational week showed a normal karyotype and low-level (30%) mosaicism of trisomy 22 by SNP array. The pregnancy was terminated at the 29th gestational week due to progressive FGR. Trisomy 22 was subsequently confirmed in skin and placental tissue. Additionally, another pregnancy (not presented in Table 2) opted for prenatal testing because of FGR, abnormal middle cerebral artery blood flow, and positive NIPT results for trisomy 9. Although invasive testing showed normal results, the pregnancy was terminated due to progressive FGR, and mosaic trisomy 9 was found in the placental tissue.

**Table 2:** Additional aberrations with clinical significance detected by SNP array analysis in 11 cases with normal karyotype

Case number	Ultrasound findings	SNP array results	Inheritance	Associated syndrome and interpretation	Outcomes
20	FGR, Echogenic bowel	arr[GRCh37] 10q11.21q11.22(42,433,738–48,006,310) × 1	De novo	LP	TOP
21	FGR, Echogenic bowel	arr[GRCh37] 10q11.22q11.23(46,252,072–51,903,756) × 3	De novo	LP	Live birth, normal development at 3-year-old follow-up
22	FGR, Increased nuchal translucency, mild tricuspid regurgitation	arr[GRCh37] 22q11.21(18,648,855–21,459,713) × 3	Maternal	22q11.2 microduplication syndrome / LP	Live birth, normal development at 3-year-old follow-up
23	FGR	arr[GRCh37] 7q11.23(72,650,120–74,154,209) × 1	De novo	Williams-Beuren syndrome / P	TOP
24	FGR	arr[GRCh37] 16q24.3(89,769,654–89,913,334) × 1	De novo	Fanconi anemia, complementation group A /P	Live birth. The infant suffered craniocerebral hemorrhage during term delivery, and rehabilitation treatment was performed for two years after birth. Her current development are close to normal
25	FGR, Increased nuchal translucency, echogenic intracardiac focus, mild tricuspid regurgitation	arr[GRCh37] Xp22.31(6,455,151–8,135,568) × 0	De novo	X-linked Ichthyosis / P	TOP
26	FGR, Echogenic intracardiac focus	arr[GRCh37] 7q11.23(72,659,097–74,207,565) × 1	De novo	Williams-Beuren syndrome/ P	TOP
27	FGR, Single umbilical artery, oligohydramnios	arr[GRCh37] 8q11.23q12.1(54,456,444–59,599,862) × 1	De novo	LP	TOP
28	FGR, Echogenic bowel, mild tricuspid regurgitation	arr[GRCh37] 6p25.3q27(203,877–170,896,644) × 2 hmz	NA	P	TOP
29	FGR^a^	Amniotic fluid at 20th gestational week: arr[hg19] 2p25.3q37.3(12,770–242,782,258) × 2–3 Cord blood at 28th gestational week: arr[GRCh37] 2p25.3p11.2(50,813–87,053,152) hmz 2q11.1q37.3(95,550,957–242,773,583) hmz	De novo	P	Premature birth, normal development at 3-year-old follow-up
30	FGR^b^	arr[GRCh37] (22) × 2 ~ 3	De novo	P	TOP

*NA* not available, *P* pathogenic, *LP* likely pathogenic, *TOP* termination of pregnancy

^a^Placental genetic study was conducted after delivery, and trisomy 2 was confirmed in placental tissue

^b^Trisomy 22 was confirmed in skin and placental tissue after delivery

### Comparison of karyotyping results

As shown in Table 3, among the four groups with different gestational age at onset, the rates of chromosome abnormality decreased with the increase of gestational age. Cases diagnosed at ≤ 24 weeks showed a 7.2% of detection rate, while in cases diagnosed after 32 weeks of gestation, there was no chromosomal aberrations. The detection rate in groups of isolated FGR, FGR with soft markers, and FGR with nonstructural abnormalities were 3.2%, 5.2%, and 4.7%, respectively (*p* > 0.05). For cases with MSS available in YMA pregnancy, there was no significant difference between high-risk and low-risk pregnancies with regard to chromosomal abnormalities by karyotyping.

**Table 3:** Distribution of chromosomal abnormalities within groups of different classifications

	**Chromosomal abnormalities (n, %)**
**Gestational age at onset**	
≤ 24 weeks (*n* = 194)	14, 7.2%
25–28 weeks (*n* = 128)	3, 2.3%
29–32 weeks (*n* = 146)	2, 1.4%
> 32 weeks (*n* = 21)	0, 0.0%
**Ultrasound findings**	
Isolated FGR (*n* = 311)	10, 3.2%
FGR with soft markers (*n* = 134)	7, 5.2%
FGR with nonstructural abnormalities (*n* = 43)	2, 4.7%
**Serological screening**	
High risk (*n* = 67)	5, 7.5%
Low risk (*n* = 149)	3, 2.7%

### Comparison of SNP array results

Among the 259 cases with normal karyotype, the detection rate of clinically relevant aberrations by CMA did not show a significant trend with gestational age at onset. However, similar to the results of karyotyping, FGR diagnosed at ≤ 24 weeks of gestation had the highest detection rate of 6.5%, and the value in FGR diagnosed at > 32 weeks was 0.0%. The incremental yield of SNP array in FGR with soft markers (9.5%) was higher than 2.2% in isolated FGR (p < 0.05), and 6.7% in FGR with nonstructural abnormalities (p > 0.05). There were only 107 cases with normal karyotype and MSS results available, and clinically significant aberrations were all observed in fetuses of high risk of MSS with a rate of 12.0%. The details were presented in Table 4.

**Table 4:** Distribution of SNP array results within groups of different classifications

Categories	Clinically significant findings (P/LP)	VOUS	LB	Normal
**Gestational age**				
≤ 24 weeks (*n* = 93)	6, 6.5%	3, 3.2%	2, 2.2%	82, 88.2%
25–28 weeks (*n* = 80)	1, 1.3%	2, 2.5%	1, 1.3%	76, 95.0%
29–32 weeks (*n* = 81)	4, 4.9%	1, 1.2%	0, 0.0%	76, 93.8%
> 32 weeks (*n* = 5)	0, 0.0%	0, 0.0%	0, 0.0%	5, 100%
**Ultrasound findings**				
Isolated FGR (*n* = 181)	4, 2.2%	1, 0.6%	5, 2.8%	171, 94.5%
FGR with soft markers (*n* = 63)	6, 9.5%	2, 3.2%	0, 0.0%	55, 87.3%
FGR with nonstructural abnormalities (*n* = 15)	1, 6.7%	0, 0.0%	1, 6.7%	13, 86.7%
**Serological screening**				
High risk (*n* = 25)	3, 12.0%	1, 4.0%	1, 4.0%	20, 80.0%
Low risk (*n* = 82)	0, 0.0%	2, 2.4%	3, 3.7%	77, 93.9%

*P* pathogenic, *LP* likely pathogenic, *VOUS* variants of uncertain significance, *LB* likely benign

### Pregnancy outcomes

Overall, 49 (10.0%) cases were lost to follow-up due to patients’ refusal or missing records. Among 439 (90.0%) cases with outcomes available, 74 (15.2%) were TOP, while 7 (1.4%) were spontaneous stillbirth or abortion, and 358 (73.6%) were live births, with 5 of them being dead during infant period.

## Discussion

This large cohort study confirmed the genetic etiology for fetuses with FGR and absent of structural malformations. Karyotyping revealed chromosomal abnormalities in 3.9% of cases. CMA detected clinically significant findings in 4.2% of 259 normal karyotype cases.

Similar to previous researches, in addition to common numerical aberrations such as trisomy 21, trisomy 18, trisomy 13, and triploidies, which were confirmed factors associated with impaired fetal growth [7, 19, 20], there were also sexual aneuploidies like 47,XXX and 47,XXY that may be considered as incidental findings. In a meta-analysis regarding CMA for fetuses with FGR and normal karyotype, Williams-Beuren syndrome (7q11.23 microdeletion), 22q11.2 microduplication syndromes, and Xp22.3 deletion syndromes were the most frequently detected pathogenic aberrations [21]. All these abnormalities were observed in our study. Additionally, two cases of UPD involving chromosome 6 and chromosome 2 respectively were observed in present study. Generally, the UPD phenotype is chromosome-specific and is dependent on paternal or maternal inheritance. For maternal UPD(6), no phenotype-genotype correlation was established, and unmasking of autosomal recessive genes in isodisomy as well as confined placental mosaicism (CPM) were speculated to be responsible for the phenotype involving FGR [22–24]. For paternal UPD(6), as observed in our study, FGR, transient neonatal diabetes mellitus, and umbilical hernia were established phenotypes induced by imprinted genes on chromosome 6 [25]. The fetus involving UPD(2)mat in our study was a complex but interesting case. The discrepancy of SNP array results between amniotic fluid and umbilical cord blood could be explained by cells of different germ layers between them. Cells in amniotic fluid belong to all three germ layers [26, 27] while cord blood cells belong to mesodermal [28]. We were not sure whether trisomy 2 cells line existed in other fetal tissues, but it’s possible to speculate that UPD(2) was a result of trisomic rescue, and CPM could be responsible for FGR. CPM refers to the phenomenon in which two or more karyotypically different cell lines are present in the placenta, whereas the fetus is usually diploid. It has been reported to increase the risk of FGR [29, 30]. So far, SNP array is the only method that enables the detection of UPD. For case 30, a low level of mosaicism trisomy 22 detected by SNP array but not karyotype may be explained by the possibility of culture selection of the normal cell line. Mosaic trisomy 22 at amniocentesis has been reported to be related to FGR [31], which was also confirmed in our study. The above findings strengthened the value of CMA, especially SNP array in FGR fetuses without structural malformation.

The association between chromosome anomalies and the onset gestational age of FGR has been frequently discussed. Most publications defined early-onset and late-onset FGR with a cutoff of 32 weeks of gestation, and suggested that chromosomal abnormalities were more commonly detected in the early-onset group [32–34]. Drummond et al.[34] did not detect any chromosomal abnormality in fetuses with isolated FGR after 28 weeks. The study by Peng et al.[33] revealed chromosomal anomaly rate of 7% in the early onset group and 1.8% in late onset group. In our cohort, cases diagnosed after 32 weeks accounted for a much smaller percentage, thus the cohort was classified into four groups according to the gestational ages at FGR diagnosis for analysis: ≤ 24 weeks, 25–28 weeks, 29–32 weeks, and > 32 weeks. Our results confirmed the inverse correlation between the gestational age and the rate of chromosomal aberrations of clinical significance. In cases diagnosed at ≤ 24 weeks of gestation, the yield of clinically significant finding by karyotyping and the yield of CMA for cases with normal karyotype were 7.2% and 6.5%, respectively, and both were higher than those in other groups. All clinically significant aberrations were found in cases diagnosed before 32 weeks of gestation. The inverse correlation could be explained by that genetic abnormalities always impairs the very initial fetal development.

FGR pregnancies were frequently categorized into structural abnormalities associated FGR and isolated FGR according to ultrasonography. In a systematic review by Sagi-Dain et al., the mean rate of chromosomal aberrations in apparently isolated FGR was estimated ranging from 0–26.3% [7]. In Monier’s study, FGR with minor ultrasound abnormalities was considered as isolate FGR, and the detection rate of anomalies with clinical significance found by CMA was estimated to be 7.5% [35]. In current study, FGR without structural malformation was further classified into isolated FGR, nonstructural abnormalities associated FGR, and soft markers associated FGR. We found similar incidence of chromosomal abnormalities detected by karyotyping among them. However, in fetuses with normal karyotype, the detection rate of clinically significant aberrations by CMA was much higher in soft marker group (9.5%) than 2.2% in isolated FGR and 5.7% in FGR with nonstructural abnormalities.

Generally, maternal serum screening (MSS) is a routine testing for pregnancies with young maternal age (< 35 years). Pregnancy-associated plasma protein A (PAPPA) and Beta-Human Chorionic Gonadotrophin (β-HCG) were synthesized by the placenta, thus were considered to be associated with placental function. They had been used to evaluate the risk of FGR in several publications [36, 37]. Here, we investigated the value of MSS in risk evaluation for chromosomal abnormalities in FGR pregnancies. The incremental yield of clinically relevant findings was 12.0% in pregnancies with high risk of MSS, significantly higher than that in low-risk pregnancies, and also much higher than 1.7% in fetuses with high-risk MSS but without structural anomalies revealed in our previous study[17]. Therefore, CMA should be strongly recommended when FGR is accompanied with high risk of MSS. However, due to the limited samples size, more evidence is required in future study to support the viewpoint.

We acknowledged the limitations that not all cases underwent karyotyping and SNP array in parallel. However, we believe that including all these cases will provide more comprehensive understanding of the fetal genetic etiology for FGR without structural abnormalities, and help patients make better decisions about whether to choose CMA testing.

In conclusion, the study demonstrated the significance of chromosomal abnormalities in FGR without structural malformation, and the superiority of SNP array compared to karyotyping in the detection of chromosomal abnormalities. Furthermore, our study also suggested that nonstructural malformation associated FGR diagnosed before 24 weeks, accompanied with soft markers, or accompanied with high-risk of MSS was related to increased risk of chromosomal aberrations.

## Data Availability

The datasets generated and/or analysed during the current study are available in the GEO repository, accession number GSE211577, https://www.ncbi.nlm.nih.gov/geo/query/acc.cgi?acc=GSE211577
